# New editorial leadership of the World Allergy Organization Journal

**DOI:** 10.1186/1939-4551-7-3

**Published:** 2014-01-30

**Authors:** Lanny J Rosenwasser

**Affiliations:** 1 Children’s Mercy Hospital, University of Missouri, Kansas City, USA; 2 School of Medicine, Kansas City 64108, USA

## 

This month the *World Allergy Organization Journal* (WAO Journal) commences its seventh year of publication and second year as an open access journal. I write this editorial from two directions, leaving the post of Editor-in-Chief and assuming the presidency of the World Allergy Organization.

The challenges involved in running a newly established, modern journal have been many and in the end rewarding. Our transition from an electronic-only subscription journal to open access was achieved last year and led to listing in PubMed Central and PubMed, which is a critical goal for a journal looking to increase its impact and visibility. The process has gone smoothly with publisher BioMed Central, and I anticipate their continued excellent support of the next editorial team.

I am also pleased to welcome the next editorial team to the WAO Journal. Alessandro Fiocchi, MD, of Hospital Bambino Gesù in Rome, Vatican City, and Erika Jensen-Jarolim, MD, of Medical University Vienna and Messerli Research Institute, Austria, are taking over the editorial leadership of the *WAO Journal*.

Professor Fiocchi brings a wide range of clinical and translational research experience to the post of Editor-in-Chief. In addition, he has been an important contributor to a variety of published material on food allergy and clinical approaches to food allergy. His outstanding leadership on two important position papers, “World Allergy Organization (WAO) Diagnosis and Rationale for Action against Cow’s Milk Allergy (DRACMA) Guidelines”, [1] and “Clinical Use of Probiotics in Pediatric Allergy (CUPPA): A World Allergy Organization Position Paper”, [2] is just one example. He is expected to make a significant impact on the quality and nature of reviews, original papers, and position papers to be published in the journal over the next year.

The appointment of Professor Erika Jensen-Jarolim as Deputy Editor will contribute to the journal’s goal of including more basic science and translational work worldwide. This will be important to maintain and solidify the journal’s march to greater impact. She has served on the journal’s Editorial Board since its inception, and brings editorial experience from a variety of medical journals to her role. Professor Jensen-Jarolim’s research experience in immunology and pathophysiology complements the clinical and translational background of Professor Fiocchi.

It was an honor to be Editor-in-Chief, following in the footsteps of giants in the field such as Alain de Weck, Gunnar Johansson, Allen Kaplan, and Johannes Ring. I hope I have maintained their standard of excellence, and I know that passing our leadership to the new team will result in the same quality we have been building.

The Regional Associate Editors and Editorial Board members all have done an outstanding job to support the journal, and it is much appreciated.

Special thanks to Managing Editor, Sofia Dorsano, for her unflagging efforts and ongoing excellent work.
